# Description of *Caurinus tlagu*, new species, from Prince of Wales Island, Alaska (Mecoptera, Boreidae, Caurininae)

**DOI:** 10.3897/zookeys.316.5400

**Published:** 2013-07-11

**Authors:** Derek S. Sikes, Jill Stockbridge

**Affiliations:** 1 University of Alaska Museum, 907 Yukon Drive, Fairbanks, AK 99775-6960, USA.

**Keywords:** *Caurinus*, Boreidae, Mecoptera, taxonomy, Prince of Wales Island, refugium

## Abstract

A new species of the cryptic, minute, wingless, and enigmatic taxon *Caurinus*, and the second for the subfamily Caurininae,is described from Prince of Wales Island in the Alexander Archipelago, Alaska. It is distinguished from its only congener, *Caurinus dectes* Russell, 1979b, which occurs 1,059 km southeast in Oregon and Washington, based on external morphology and sequences of the mitochondrial gene cytochrome oxidase II. These two species are probably evolutionary relicts – the only known members of a clade dating to the Late Jurassic or older.

## Introduction

Russell (1979a, b, 1982) described the monotypic subfamily Caurininae, genus and species *Caurinus dectes*, known only from Oregon and Washington, and later described by Beutel et al. (2008) as “arguably one of the most bizarre and cryptic species of Mecoptera and endopterygote insects.” Indeed, members of the genus do not key to any order in most keys to insect orders because they lack a produced rostrum, typical of the order Mecoptera, and lack the diagnostic traits that would place them within *any* insect order containing flightless adults with rudimentary or vestigial wings. However, they do share with members of the family Boreidae a very distinctive wing morphology and sexual dimorphism in which the adult females are nearly wingless while the males bear shortened scissor-like wings, useless for flight, that bear spines for grasping females during mating. The placement of *Caurinus* within the Mecopteran family Boreidae as the sister taxon to the Boreinae (*Boreus* 26 spp., *Hesperoboreus* 2 spp. [Penny 2013]), is apparently well established based on morphological study (Russell 1979a, b, Beutel et al. 2008, Friedrich et al. 2013) and molecular phylogenetics (Whiting 2002). However, despite recent efforts, the genus remains enigmatic due to its preponderance of plesiomorphic and autapomorphic traits (Beutel et al. 2008). The close relationship of the Mecoptera with the fleas, order Siphonaptera, is of particular evolutionary interest (Grimaldi and Engel 2005, Whiting 2002, Trautwein et al. 2012).

It was therefore with some excitement that we began accumulating *Caurinus* specimens from a large sampling project on the northern end of Prince of Wales Island, Alaska, some 1,059 km from the known range of *Caurinus dectes* Russell. Herein we describe this new species.

## Materials and methods

Collections. Specimens will be deposited in the following collections:

CAS California Academy of Sciences, San Francisco, California, USA. (Norm Penny)

MTEC Montana Entomology Collection, Bozeman, Montana, USA. (Michael Ivie)

OSAC Oregon State Arthropod Collection, Oregon State University, Corvallis, Oregon, USA. (David R. Maddison)

PMJ Phyletisches Museum, Jena, Germany (Rolf G. Beutel)

SEMC Snow Entomological Museum, University of Kansas, Lawrence, Kansas, USA. (George Byers)

UAM University of Alaska Museum Insect Collection, University of Alaska, Fairbanks, Alaska, USA. (Derek S. Sikes)

USNM National Museum of Natural History, Washington D.C., USA. (Ollie Flint)

**Morphological methods.** Images of *Caurinus tlagu* were captured using a Leica DFC425 camera mounted on a Leica MZ16 stereomicroscope in combination with Leica Application Suite © software v.3.8.0. Images were edited using Adobe Photoshop v.7 to remove the background and lighten the images. Observations were made with a Leica MZ16 stereomicroscope (7.1×–115× magnification, 1x planapochromatic objective/10× eyepieces, max resolution 420 Lp/mm, Leica Microsystems (Switzerland) Ltd.). Measurements were made using an ocular micrometer in the MZ16 scope at 50×. Five *Caurinus tlagu* specimens were prepared for scanning electron microscopy (SEM) using a Tousimis Samdri-790 Critical Point Dryer and sputter (gold) coating with a Ladd coating unit. The scanning electron micrographs were taken with a ISI-SR-50 SEM and the digital imaging program Iridium Digital Imaging System. In addition to the images included herein, many more SEMs and habitat photos are associated with their specimen records via our online database Arctos (http://arctos.database.museum/saved/Caurinus-spp).

**Taxon sampling.** Two Mecoptera COII sequences from GenBank were used as outgroups: *Boreus westwoodi* Hagen (EU335963.1) and *Boreus hyemalis* (L.) (AF423998.1). *Boreus* species were chosen because they share the family assignment of Boreidae with *Caurinus* and therefore should be more closely related to *Caurinus* than any other genus in GenBank. The single *Caurinus dectes* COII sequence on GenBank (AF424001.1) was initially included (and its existence drove our desire to sequence COII rather than the more common gene COI), but later excluded due to it being suspected of errors (see below). One of the five Alaskan *Caurinus* specimens had ambiguous reads in both directions for its COII sequence, possibly due to co-amplification of a nuclear copy. We excluded this sequence from analysis.

*Caurinus dectes* specimens were provided by L. Russell. Seven specimens from Lewis County, Washington, collected in 1978 were provided for morphological study and 12 larval and 11 adult specimens from 2012 collections made in Benton and Tillamook Counties, Oregon, for DNA analysis (Table 1). Our collecting efforts on Prince of Wales Island have yielded 37 specimens (18 males, 19 females) of *Caurinus tlagu* (see Collecting methods below, Table 1). Additional, non-type specimens are likely to be found as sampling progresses. These specimens will be archived in UAM and recorded in our online database, Arctos.

**DNA sequencing**. Adult specimens and larvae designated for DNA extraction were stored at -70°F in cryovials containing 100% EtOH. Specimen data are presented in Table 1. DNA was extracted from whole bodies of five adult specimens from the Alaskan population and from seven whole bodies of the Oregon larvae. During the extraction process, specimens were opened with a pin prick to allow full extraction of DNA from soft tissues. After extraction was complete, specimens were soaked overnight in 70% EtOH to stop further deterioration of specimen exoskeletons in order to preserve them for future morphological study. Extractions were performed using a Qiagen DNeasy© blood and tissue extraction kit which was used according to the spin-column protocol for animal tissues. To amplify the COII gene, the following primer pair was used: forward COII-2a (ATAGAKCWTCYCCHTTAATAGAACA) and reverse COII-9b (GTACTTGCTTTCAGTCATCTWATG) taken from Whiting (2002).

Upon completion, extraction success was tested using a nano-drop spectrophotometer. DNA concentrations were (0.5–4.0 ng/µL). Primers were diluted at a relatively high concentration of 10µM in accordance with Whiting (2002). PCR was performed using the following 25µl PCR-mix: 12.5µl GoTaq DNA polymerase, 1µl each of the forward and reverse primers, 1µl Mg+, 9.75µL DNA-grade distilled water and 1µL template DNA. The following cycling regime was applied: (1) 1 min at 95°C, followed by (2) 35 cycles of 1 min at 95°C, 1 min at 59°C, and 1 min at 72°C, and (3) a final extension of 7 min at 72°C. Amplification success and correct band length was confirmed visually on an agarose gel stained with ethidium bromide. Bi-directional sequencing was performed at the University of Washington’s High Throughput Genomics Unit.

**Alignment.** Sequences were aligned using CodonCode Aligner v4.0.4 (http://www.codoncode.com/aligner/) and proofread by eye with reference to codon position and the inferred amino acid sequence based on Liu and Beckenbach (1992). Alignment was without difficulty due to the absence of indels within the protein-coding sequence. MacClade was used to produce a consensus of forward and reverse reads (Maddison and Maddison 2005).

**Model Selection.** JModelTest v2.1.3 (Darriba et al. 2012, Guindon and Gascuel 2003) was used to determine the best fitting model among 88 available for testing. The AIC, BIC and DT all chose the model HKY+G as the best fit for the data.

**Analysis.** Bayesian analyses were conducted using MrBayes v3.2 (Ronquist and Huelsenbeck 2003) under the HKY+G model with default priors. Two simultaneous MCMC runs with four chains each (3 hot and 1 cold) were performed for 10 million generations and sampled every 1,000 steps discarding a burnin of 25%. To evaluate whether the MCMC analysis had reached stationarity, trace files were examined in Tracer v1.5 (Rambaut and Drummond 2003). These showed signs of good mixing and had plateaued at equal values. The average standard deviation of split frequencies between the two runs had dropped below 0.01 by 12% of the 10M step run, also indicating both runs had converged. Maximum Likelihood analyses were conducted using Garli v.2.0.1019 (Zwickl 2006) under the HKY+G model with 1000 non-parametric bootstrap search replicates in addition to a non-bootstrap analysis of 100 search replicates from random starting trees.

**Figure 1.**
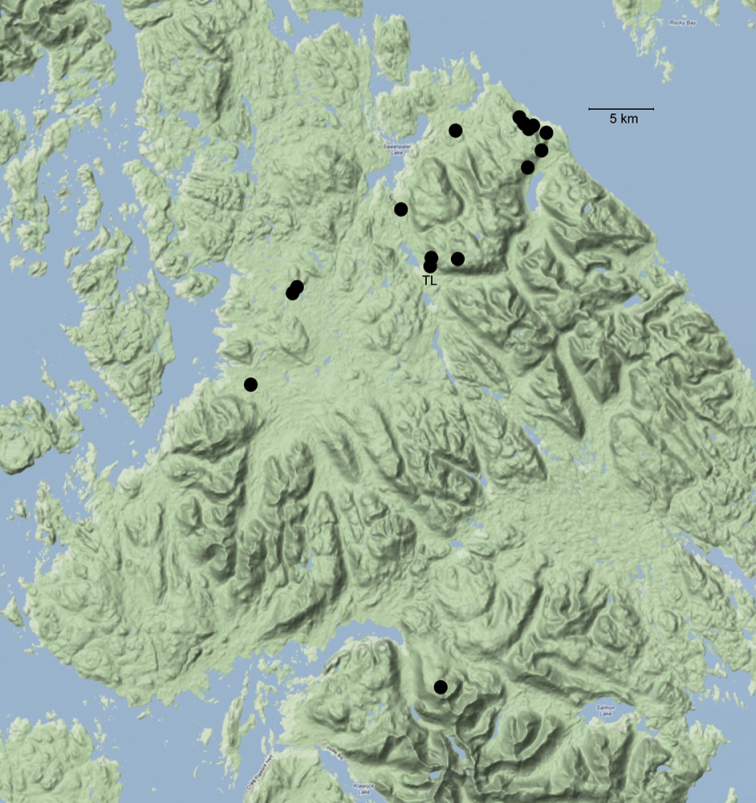
Sixteen sites at which *Caurinus tlagu* specimens were found, north end of Prince of Wales Island, Alaska. Table 1 lists site and specimen data, also available online at http://arctos.database.museum/saved/Caurinus-AK. TL = type locality.

**Collecting methods and results.** Specimens of this new species were collected primarily using pitfall traps and Berlese funnels (Table 1) as part of our four year, ongoing project investigating forestry practices in the Tongass National Forest (Fig. 1). Two specimens were caught in a very different habitat in pitfall traps set on a transect of 20 traps spaced 100m apart in a treeless alpine zone (917m elevation) near Black lake, Prince of Wales Isl., with tundra-alpine-heath vegetation (e.g. *Harrimanella stelleriana*, *Luetkea pectinata*, *Rhytidiadelphus loreus*). This collection was part of a rapid biotic assessment of Southeast Alaska alpine zones (Fig. 1A) and was located 45 km southwest of the Coffman Cove collection sites. Pitfall traps consisted of paired (Coffman Cove) or single (alpine) plastic cups 8.3 cm in diameter and 7.5 cm deep filled 1/2–2/3 with non-toxic propylene glycol based antifreeze, Sierra © brand (Coffman Cove), or soapy water (alpine) with rain-roofs ~3 cm from the ground above the traps. Traps were emptied once every two weeks (Coffman Cove) or daily (alpine zone). Paired traps were 30cm apart with a plastic ruler embedded in the ground between them to act as a barrier to divert arthropods into the traps. As part of the Tongass sampling, BioQuip © collapsible Berlese funnels were used with ~ 1m^2^ of leaf/moss litter sifted prior to running under 40 watt bulbs for 48h. These methods resulted in 37 specimens collected. However, incredible effort was involved. A total of 1,136 pitfall trap and 284 Berlese samples were processed from 2010 and 2011 that have generated 10,218 beetle specimens to date. The alpine sampling involved 83 pitfall trap samples, which yielded two *Caurinus* specimens. Twenty-six *Caurinus* specimens were captured in pitfall traps, ten in Berlese funnels, and one, surprisingly, in a Lindgren funnel. Great care was taken to ensure pitfall trap rims were at or below the level of the ground – certainly an important factor when trapping an animal ~ 2 mm in size.

**Table 1.** Specimen data (n=50 lots). Also available online at http://arctos.database.museum/saved/Caurinus-spp via Arctos. Geocoordinates are in WGS84 datum. PoW = Prince of Wales Island. * = holotype male *Caurinus tlagu*, with genitalia everted and COII gene sequenced. All other *Caurinus tlagu* specimens are paratypes. W-screen = wet screen, Hab. = habitat. Habitat type codes: T2 = thinned secondary growth, 2= young secondary growth (unthinned), 2o = old (80yr) secondary growth, CC = clearcut, CCe = clearcut / forest ecotone, OG = old growth, AH = alpine heath. Date1 and Date2 = start and stop dates for trap samples.

**Catalog Number**	**Species**	**State**	**Locality**	**Hab.**	**Method**	**Date1**	**Date2**	**Latitude**	**Longitude**	**+/-** **(m)**	**sex / stage**
UAM:Ento:121022	*Caurinus tlagu*	Alaska	PoW Is. Coffman Cv	T2	pitfall	4/27/10	5/15/10	55.9795, -132.86256		101	male
UAM:Ento:121023	*Caurinus tlagu*	Alaska	PoW Is. Coffman Cv	T2	Berlese	5/13/10		55.9795, -132.86256		101	female
UAM:Ento:135818	*Caurinus tlagu*	Alaska	PoW Is. Coffman Cv	T2	pitfall 4	5/14/10	5/28/10	55.9795, -132.86256		101	male
UAM:Ento:159146	*Caurinus tlagu*	Alaska	PoW Is. Coffman Cv	T2	pitfall 2	7/14/10	7/26/10	55.9795, -132.86256		101	male
UAM:Ento:202339	*Caurinus tlagu*	Alaska	PoW Is. Coffman Cv	T2	pitfall 4	5/18/11	5/31/11	55.9795, -132.86256		101	female, male
UAM:Ento:204005	*Caurinus tlagu*	Alaska	PoW Is. Coffman Cv	T2	Berlese 2	6/14/11		55.9795, -132.86256		101	female, male
UAM:Ento:229946	*Caurinus tlagu*	Alaska	PoW Is. Coffman Cv	T2	pitfall 4	7/27/11	8/7/11	55.9795, -132.86256		101	female
UAM:Ento:229944	*Caurinus tlagu*	Alaska	PoW Is. Hatchery Ck.1	OG	Berlese	8/9/11		55.92444, -132.93938		4	female
UAM:Ento:142985	*Caurinus tlagu*	Alaska	PoW Is. Hatchery Ck.4	OG	pitfall 2	5/14/10	5/30/10	55.88602, -132.8607		11	female
UAM:Ento:142986 *	*Caurinus tlagu*	Alaska	PoW Is. Hatchery Ck.4	T2	pitfall 3	5/30/10	6/14/10	55.88433, -132.89734		26	male
UAM:Ento:204239	*Caurinus tlagu*	Alaska	PoW Is. Hatchery Ck.4	OG	pitfall 2	5/31/11	6/14/11	55.88602, -132.8607		11	male
UAM:Ento:217990	*Caurinus tlagu*	Alaska	PoW Is. Hatchery Ck.4	OG	pitfall 3	6/28/11	7/12/11	55.88602, -132.8607		11	male
UAM:Ento:221708	*Caurinus tlagu*	Alaska	PoW Is. Hatchery Ck.4	2	Berlese 5	7/27/11		55.88285, -132.89795		27	female
UAM:Ento:203237	*Caurinus tlagu*	Alaska	PoW Is. Luck Lk. 1 Rd.	OG	pitfall 4	5/24/11	6/5/11	55.97805, -132.75456		10	female
UAM:Ento:216180	*Caurinus tlagu*	Alaska	PoW Is. Luck Lk. 1 Rd.	OG	pitfall 4	6/21/11	7/6/11	55.97805, -132.75456		10	male
UAM:Ento:154335	*Caurinus tlagu*	Alaska	PoW Is. Luck Lk. 2 Rd.	OG	pitfall 1	7/8/10	7/30/10	55.96855, -132.75615		10	female
UAM:Ento:203238	*Caurinus tlagu*	Alaska	PoW Is. Luck Lk. 2 Rd.	OG	pitfall 3	5/24/11	6/5/11	55.96855, -132.75615		10	male
UAM:Ento:159119	*Caurinus tlagu*	Alaska	PoW Is. Luck Lk. 3 Rd.	OG	Berlese 4	7/29/10		55.95347, -132.7708		5	female
UAM:Ento:203239	*Caurinus tlagu*	Alaska	PoW Is. Luck Lk. 3 Rd.	OG	Berlese 1	6/5/11		55.95347, -132.7708		5	female
UAM:Ento:133943	*Caurinus tlagu*	Alaska	PoW Is. Luck Point	CC	Berlese 2	5/21/10		55.98497, -132.787		25	male
UAM:Ento:159120	*Caurinus tlagu*	Alaska	PoW Is. Luck Point	CC	pitfall 1	7/9/10	8/1/10	55.97953, -132.77156		24	female
UAM:Ento:167053	*Caurinus tlagu*	Alaska	PoW Is. Luck Point	CC	pitfall 1	8/1/10	8/11/10	55.97953, -132.77156		24	male
UAM:Ento:203011	*Caurinus tlagu*	Alaska	PoW Is. Luck Point	T2	pitfall 1	5/23/11	6/5/11	55.98261, -132.77986		6	female
UAM:Ento:229942	*Caurinus tlagu*	Alaska	PoW Is. Luck Point	CC	pitfall 1	8/2/11	8/9/11	55.97953, -132.77156		24	female
UAM:Ento:229943	*Caurinus tlagu*	Alaska	PoW Is. Luck Point	CC	Lindgren	8/2/11	8/9/11	55.97939, -132.77216		25	male
UAM:Ento:121024	*Caurinus tlagu*	Alaska	PoW Is. Staney Ck.	CCe	pitfall	4/27/10	5/15/10	55.87126, -133.06697		5	male
UAM:Ento:202344	*Caurinus tlagu*	Alaska	PoW Is. Staney Ck.	CC	pitfall 3	5/16/11	5/31/11	55.872, -133.06523		26	male
UAM:Ento:229945	*Caurinus tlagu*	Alaska	PoW Is. Staney Ck.	OG	pitfall 4	7/12/11	7/27/11	55.79901, -133.11782		20	male
UAM:Ento:230091	*Caurinus tlagu*	Alaska	PoW Is. Staney Ck.	OG	pitfall 2	5/14/12	5/28/12	55.79901, -133.11782		20	female
UAM:Ento:231726	*Caurinus tlagu*	Alaska	PoW Is. nr Black Lk	AH	pitfall	7/9/11	7/10/11	55.58818, -132.88881		2	male
UAM:Ento:231727	*Caurinus tlagu*	Alaska	PoW Is. nr Black Lk	AH	pitfall	7/9/11	7/10/11	55.58818, -132.88881		2	female
UAM:Ento:235023	*Caurinus tlagu*	Alaska	PoW Is. Hatchery Ck.4	OG	pitfall	5/15/12	5/28/12	55.88602, -132.8607		11	female
UAM:Ento:235024	*Caurinus tlagu*	Alaska	PoW Is. Luck Point	CC	Berlese	5/31/12		55.98497, -132.787		25	female
UAM:Ento:235025	*Caurinus tlagu*	Alaska	PoW Is. Luck Lk. 1 Rd.	OG	pitfall	5/16/12	5/31/12	55.97805, -132.75456		10	female
UAM:Ento:235026	*Caurinus tlagu*	Alaska	PoW Is. Luck Lk. 3 Rd.	OG	Berlese	5/22/12		55.95347, -132.7708		5	male
UAM:Ento:230088	*Caurinus dectes*	Oregon	Mary’s Peak	2o	w-screen Berlese	10/30/12		44.50413, -123.55125		5000	female, male
UAM:Ento:228446	*Caurinus dectes*	Oregon	Marys Peak	2o	w-screen	5/11/12		44.50413, -123.55125		5000	larva
UAM:Ento:228447	*Caurinus dectes*	Oregon	Marys Peak	2o	w-screen	5/11/12		44.50413, -123.55125		5000	larva
UAM:Ento:228448	*Caurinus dectes*	Oregon	Marys Peak	2o	w-screen	5/11/12		44.50413, -123.55125		5000	larva
UAM:Ento:228449	*Caurinus dectes*	Oregon	Marys Peak	2o	w-screen	5/11/12		44.50413, -123.55125		5000	larva
UAM:Ento:228450	*Caurinus dectes*	Oregon	Marys Peak	2o	w-screen	5/11/12		44.50413, -123.55125		5000	larva
UAM:Ento:228451	*Caurinus dectes*	Oregon	Marys Peak	2o	w-screen	5/11/12		44.50413, -123.55125		5000	larva
UAM:Ento:228452	*Caurinus dectes*	Oregon	Marys Peak	2o	w-screen	5/11/12		44.50413, -123.55125		5000	larva
UAM:Ento:228453	*Caurinus dectes*	Oregon	Marys Peak	2o	w-screen	5/11/12		44.50413, -123.55125		5000	larva
UAM:Ento:228454	*Caurinus dectes*	Oregon	Marys Peak	2o	w-screen	5/11/12		44.50413, -123.55125		5000	larva
UAM:Ento:228455	*Caurinus dectes*	Oregon	Marys Peak	2o	w-screen	5/11/12		44.50413, -123.55125		5000	larva
UAM:Ento:228456	*Caurinus dectes*	Oregon	Marys Peak	2o	w-screen	5/11/12		44.50413, -123.55125		5000	larva
UAM:Ento:228457	*Caurinus dectes*	Oregon	Marys Peak	2o	w-screen	5/11/12		44.50413, -123.55125		5000	larva
UAM:Ento:228458	*Caurinus dectes*	WA	Lewis Co.	2o	Berlese	5/6/78		46.62848, -122.27701		5000	male, female
UAMObs:Ento:228643	*Caurinus dectes*	Oregon	Cape Lookout	2o	w-screen	11/6/12		45.33954, -123.99289		5000	male
UAM:Ento:234931	*Caurinus dectes*	Oregon	Cape Lookout	2o	w-screen	11/6/12		45.33954, -123.99289		5000	male

**Figure 2.**
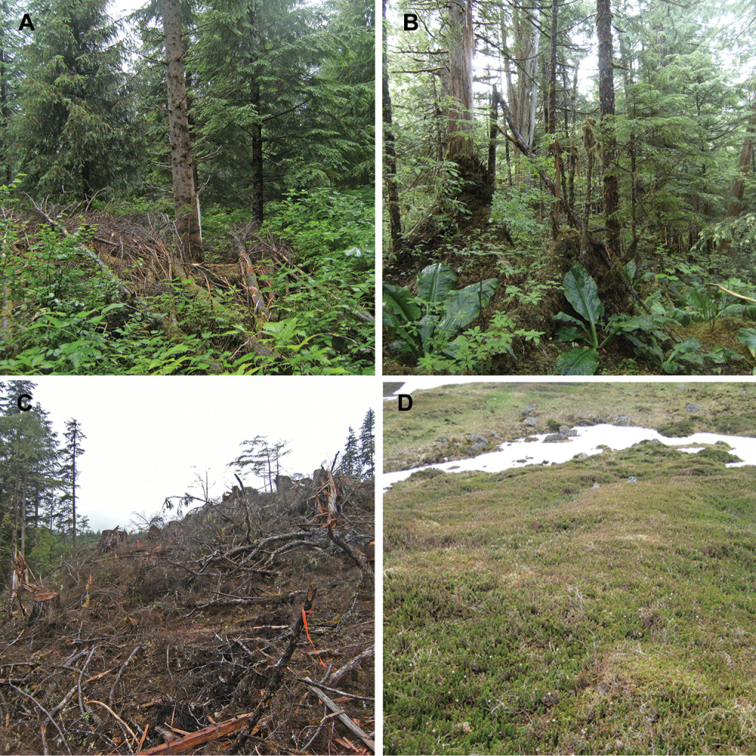
Habitats of *Caurinus tlagu* **A** Habtiat of type locality, thinned secondary growth with 18 ft. spacing between trees, 55.88433, -132.89734 **B** example of old growth habitat in which specimen UAM:Ento:204239 was found, 55.88602,-132.8607 **C** example of clearcut, a habitat type in which seven specimens were found, 55.872, -133.06523 **D** example of treeless, alpine heath – tundra in which two specimens were found, 55.58818, -132.88881.

The majority of specimens (35/37) were collected in perhumid rainforest dominated by Sitka spruce (*Picea sitchensis*), western hemlock (*Tsuga heterophylla*), lodgepole pine (*Pinus contorta* var. *contorta*), Alaska yellow cedar (*Chamaecyparis nootkatensis*), red cedar (*Thuja plicata*), and red alder (*Alnus rubra*) (Fig. 2). Of 24 sites sampled in the Tongass National Forest project, *Caurinus* was found in 14 sites. Fifteen specimens were found in six of six sampled old growth sites, eleven in three of six sampled thinned secondary growth sites, seven in four of six sampled clear cuts, and one in one of six sampled unthinned secondary growth sites. One additional specimen was found in an ecotone next to a clear cut that was not part of the 24 structured sampling sites. The null hypothesis of *Caurinus* being equally trappable in all four habitat types: old growth, thinned secondary growth, unthinned secondary growth, and clear cuts, (ignoring the ecotone), is rejected (Chi^2^ = 12.59, df=3, P=0.0056). These animals are less trappable in unthinned secondary growth sites than expected under the null, and more trappable in old growth and thinned secondary growth sites than expected under the null.

Although boreids are considered winter active insects, our projects were restricted to the summer months. We caught *Caurinus* more or less evenly throughout the period of sampling (mid May – mid August) (Table 1).

## Results from molecular analyses

**DNA sequence characteristics.** The final alignment of the DNA sequences (11 *Caurinus* sequences, 2 outgroup *Boreus* sequences) was 639 base pairs long with 491 constant sites, 21 variable but parsimony-uniformative sites, and 127 parsimony informative sites. Among the *Caurinus* sequences there were 604 constant sites and 35 parsimony informative sites. Of these 35 variable sites between the *Caurinus* species, 34 were binary with all specimens of each species sharing the same base differing from the other species. As expected, most (29) of these variable sites were third codon positions, with six variable first codon position sites, and zero variable second codon position sites. The null hypothesis of homogeneity of base frequencies across taxa was not rejected by a Chi-square test performed in PAUP*4.0b10 (Chi^2^=27.5, df=36, P=0.85) (Swofford 2003). These sequences are available from Genbank (accession numbers KF282717 through KF282727) and the aligned NEXUS and tree files are available from TreeBase (http://purl.org/phylo/treebase/phylows/study/TB2:S14415) under study Accession number 14415.

The *Caurinus* species are 98.5% identical in their inferred COII amino acid sequences (209 of 212 amino acids are identical). The three amino acid replacements are as follows: The 113^th^ site of the amino acid translation is an Alanine (nonpolar) shared by all seven *Caurinus dectes* specimens but is a Threonine (polar) in all five *Caurinus tlagu* specimens; at the 114^th^ site an Aspartic acid (acid polar) shared by all seven *Caurinus dectes* specimens is a Asparagine (polar) in all five *Caurinus tlagu* specimens; and at the 148^th^ site an Isoleucine (nonpolar) shared by all seven *Caurinus dectes* specimens is a Valine (nonpolar) in all five *Caurinus tlagu* specimens.

All seven *Caurinus dectes* share identical COII nucleotide sequences whereas only three of the *Caurinus tlagu* share identical sequences, the fourth *Caurinus tlagu* differs at one site (0.156% divergent) from the other three *Caurinus tlagu*. The two *Caurinus* species are 5.44% divergent from each other (uncorrected “p” distance). The two outgroup species are 3.9% divergent from each other, and 21% (*Boreus hyemalis*) to 20% (*Boreus westwoodi*) divergent from *Caurinus*. The COII GenBank record of *Caurinus dectes* (AF424001.1) is 21.7% divergent from the seven *Caurinus dectes* we sequenced. Using the parameter values from the Garli analysis (see below) to set the HKY+G model in PAUP*4.0b10 allowed the estimation of distances corrected for multiple hits: the two *Caurinus* species are 7.17% divergent from each other. The two outgroup species are 5.6% divergent from each other, and 106.7% (*Boreus hyemalis*) to 103.5% (*Boreus westwoodi*) divergent from *Caurinus*.

**Bayesian Analysis.** Tracer reported auto-correlation times of 1027 and 1015 for the two runs with Effective Sample Sizes for all parameters of each run above 7000 (with samples from both runs combined, the ESS of each parameter was above 15,000). Parameter estimates of both runs combined were as follows: the harmonic mean of the estimated marginal likelihood was –1515.7, tree length 0.692, the transition/transversion rate ratio (kappa) 6.59, pi(A) 0.356, pi(C) 0.151, pi(G) 0.102, and pi(T) 0.391 with the alpha shape parameter at 0.258.

**Figure 3.**
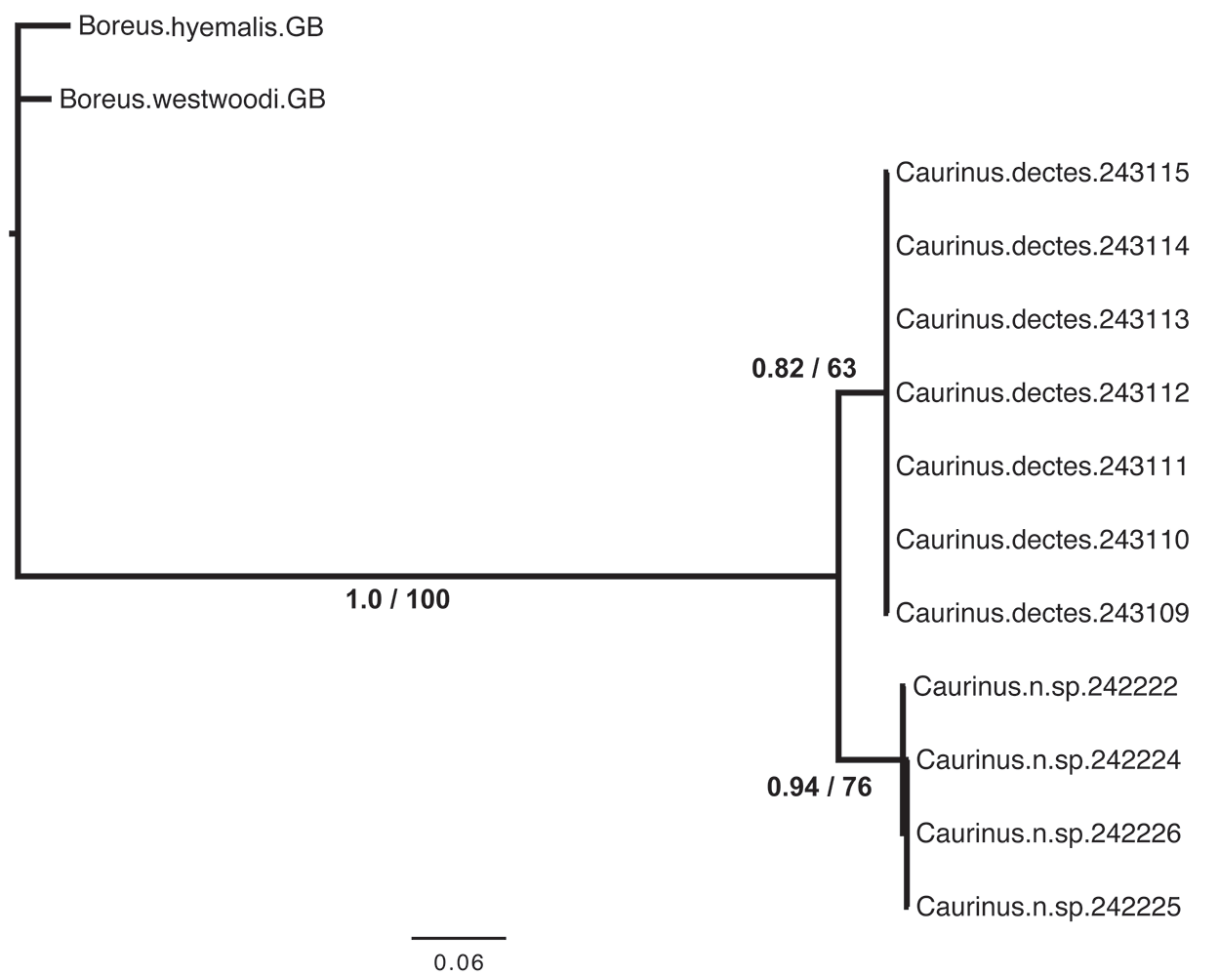
Inferred phylogeny from Bayesian analysis. Each terminal is a single specimen with the UAM cryovial barcode of its DNA extraction indicated by a six digit number. Branch support is indicated as estimated posterior probability from the Bayesian analysis first and maximum-likelihood bootstrap percentages second. Branch lengths are proportional to the number of substitutions per site as reconstructed by MrBayes 3.2. Specimen 242224 is the holotype of *Caurinus tlagu* http://arctos.database.museum/guid/UAM:Ento:142986. The remaining three *Caurinus tlagu* specimens correspond to the following paratypes in Table 1: 242222 (UAM:Ento:135818), 242225 (UAM:Ento:159119), and 242226 (UAM:Ento:154335).

**Garli Analysis.** The 1000 bootstrap replicate analysis resulted in similarly strong branch support values as the Bayesian analysis (Fig. 3). One hundred non-bootstrap replicates were completed, the best tree of which was found in 96 of the searches and was identical in topology to the Bayesian tree (Fig. 3) with a -lnL of 1476.75, tree length of 0.858, and parameter values of: K parameter 8.789, ti/tv 3.321, pi(A) 0.3596, pi(C) 0.1481, pi(G) 0.0991, and pi(T) 0.3933 with the alpha shape parameter at 0.1733.

Both the Bayesian and maximum likelihood analyses found strong support for reciprocal monophyly of both *Caurinus* species (Fig. 3).

## Systematics

### Caurinus tlagu

Sikes & Stockbridge sp. n.

urn:lsid:zoobank.org:act:BFFF780A-737D-4187-8539-32270D80D4C5

http://species-id.net/wiki/Caurinus_tlagu

#### Holotype.

Male (in UAM), here designated, labeled “USA: Alaska, Prince of Wales Is. Hatchery Ck.4, 30 May-14 June 2010, 55.88433°N, 132.89734°W ± 26m, 82m elev., thinned secondary growth with 18 ft. spacing between trees, pitfall 3, J. Stockbridge, C. Bickford”, / “HOLOTYPE *Caurinus tlagu* Sikes & Stockbridge 2013 UAM:Ento:142986” [red paper]. http://dx.doi.org/10.7299/X7GH9J4M

#### Paratypes.

36 Specimens (Table 1). The following 17 paratypes will be deposited in the collections indicated: male UAM:Ento:159146, female UAM:Ento:142985, female UAM:Ento:235025 (CAS); male UAM:Ento:229945, female UAM:Ento:235024, female UAM:Ento:229942 (OSAC); male UAM:Ento:235026, female UAM:Ento:203239, female UAM:Ento:203011 (PMJ); male UAM:Ento:167053, female UAM:Ento:229944, female UAM:Ento:235023 (SEMC); male UAM:Ento:217990, female UAM:Ento:221708, female UAM:Ento:159120 (USNM); male UAM:Ento:229943, female UAM:Ento:230091 (MTEC), and the 19 remaining in UAM.

#### Type Locality.

USA: Alaska, Prince of Wales Is. Hatchery Ck, 55.88433°N, 132.89734°W ± 26m, 82m elev. (Fig. 1, 2A).

#### Measurements.

Restricted to specimens with retracted genitalia (3 males, 10 females), length, min. – max., mean ± SD: male 1.58–2.02, 1.74 ± 0.24 mm, female 1.64 – 2.00, 1.79 ± 0.13 mm.

#### Diagnosis.

**Figure 4.**
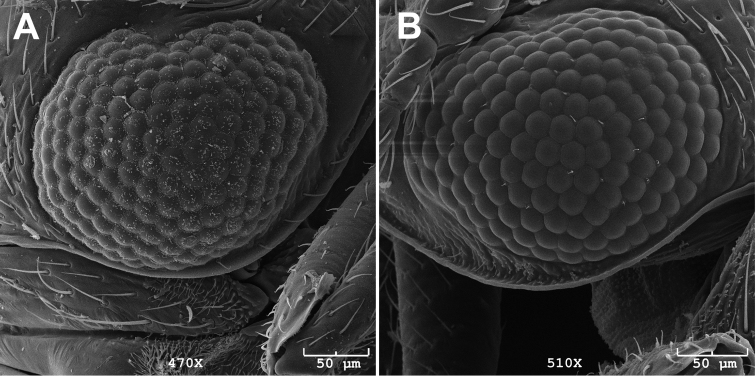
Eye of **A** male *Caurinus dectes* (UAM:Ento:230088) showing 38 ommatidia around circumference of right eye, dorsal is to the left, and **B** male *Caurinus tlagu* (UAM:Ento:202344) showing 35 ommatidia around circumference of left eye, dorsal is to the right. Scale bar = 50 µm.

**Figure 5.**
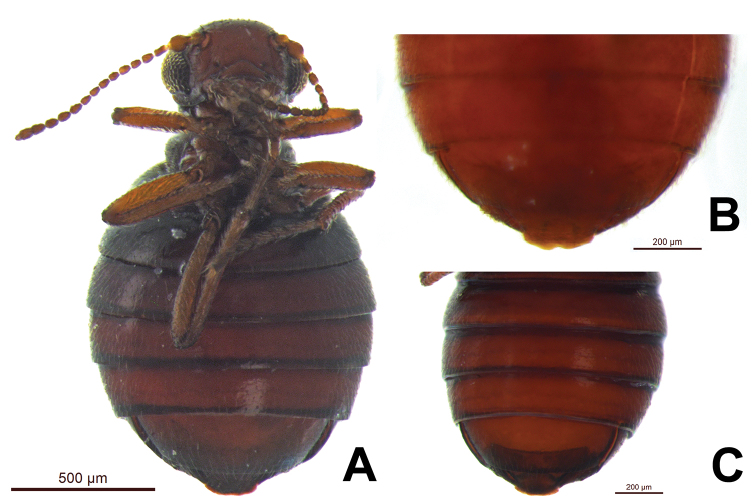
**A** ventral view of female *Caurinus tlagu* (UAM:Ento:203239) showing 8^th^ sternum with shallow median emargination / notch, scale bar = 500 µm **B** ventral view of abdomen of female *Caurinus dectes* (UAM:Ento:228458) showing 8^th^ sternum with a pronounced notch, scale bar = 200 µm **C** ventral view of abdomen of female *Caurinus tlagu* (UAM:Ento:203011) showing 8th sternum with shallow emargination / notch, scale bar = 200 µm.

Circumference of eye of males comprises 31-35 (n=3) ommatidia (*Caurinus dectes* males have 38–39, n=3). Scanning electron microscope-level resolution is required to obtain reliable counts (Fig. 4). Female 8^th^ sterna without a median notch (n=10), or with a shallow median notch (n=5) (Fig. 5A, C, 6C, D). *Caurinus dectes* females have a shallow median notch or a pronounced median notch (Fig. 5B, see also Russell [1979b] fig. 10). This is visible at 40× and higher magnification.

**Figure 6.**
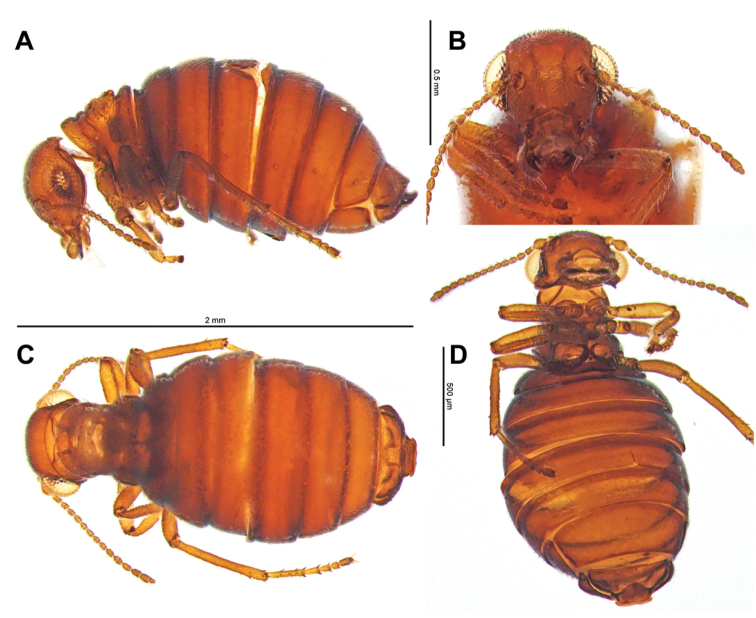
Female *Caurinus tlagu* (UAM:Ento:159119) that had been cleared in KOH. **A** lateral view (broken abdomen), scale bar = 2 mm **B** face, scale bar = 0.5 mm **C** dorsal view, scale bar = 2 mm **D** ventral view, scale bar = 0.5 mm.

#### Description.

**Figure 7.**
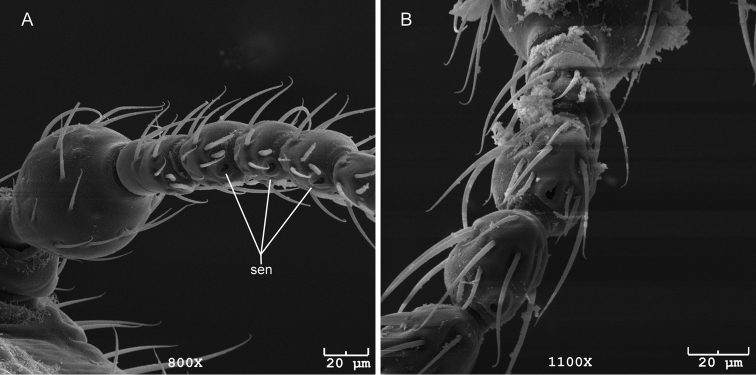
Base of *Caurinus* antenna showing sensilla on antenomeres 4, 5, and 6. **A** female *Caurinus dectes* (UAM:Ento:230088), **B** female *Caurinus tlagu* (UAM:Ento:203237); sen = sensilla, scale bars = 20 µm.

**Figure 8.**
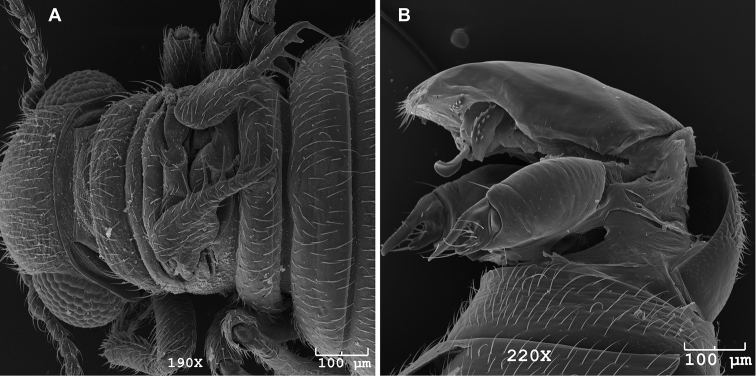
SEM images of male *Caurinus tlagu* (UAM:Ento:204239), scale bars = 100 µm **A** dorsal view showing wings **B** evertedgenitalia showing paired gonostyles, oblique lateral view.

Body length 1.5–2.3 mm, flea-like in lateral view, color reddish brown, sparsely pubescent, strongly sclerotized (Fig. 6). Rostrum absent or reduced. Clypeolabral suture present. Clypeus divided into post and anteclypeus. Penultimate maxillary palpomere enlarged and club shaped. Antennal insertion lateral, widely separated. Ocelli absent. Antennae with sixteen antennomeres and a single countersunk sensilla on antennomeres 4, 5, and 6 (Fig. 7). Mandible with two subapical teeth (Fig. 6B). Male forewings extend to end of first abdominal segment, with six bristles (Fig. 8A), hindwings absent. Female forewings pad-like, hindwings absent. Tarsi five segmented, tarsal claws present. Pilosity absent. Abdomen widest at segments 4 and 5, segments 2-6 fused, annular. Male 8th tergum and sternum not fused. Male 9th tergum and sternum not fused. Genitalia normally concealed in both sexes. Male gonostyles flattened, deeply incised (Fig. 8B).

#### Variation.

One male (UAM:Ento:231726) has 7 bristles on its right wing, as a result of a very small extra basal bristle, and six on its left.

#### Geographic Distribution and Habitat.

This species is only known from the northern half of Prince of Wales Island within a region about 45 km in size (Fig. 1). It was collected in forest habitat of various stages: old growth, secondary growth (thinned and unthinned), and young clear cuts; in addition to two specimens caught in alpine heath habitat and one in an ecotone of clearcut / secondary forest. The species is not restricted to lowland forests, nor to old growth forests.

#### Etymology.

“*Tlagu*”, pronounced “tlu-gu”, is derived from the Alaska Native tribal language Tlingit meaning “ancient, forever” (Crippen 2013) or “old, from the past” (Edwards 2009). Bierhorst (1985) provided this elaboration: “Among the Tlingit, for example, there are two kinds of stories, *tlagu* (of the long ago) and *ch’kalnik* (it really happened).” We name this species in honor of the place it occurs, its people, and history, in addition to the apparent great age of the genus *Caurinus*.

## Discussion

Diagnostic characters were not easily found. These species are very similar phenotypically. The use of ommatidia counts around the circumference of the eyes of males (females we examined overlapped in these counts) is certainly not an ideal character because it is limited to one sex and requires SEM imaging to obtain accurate counts. In part because of this difficulty, and the rarity of specimens, our sample sizes for the assessment of this character are suboptimal. Despite these small sample sizes (n=3 for each species) the means differ significantly based on an unpaired, two-tailed student’s t-test (p = 0.0142). We hope that ongoing morphological study of the Mecoptera by Rolf Beutel and others (e.g. Beutel et al. 2008) will better document variation between and within these *Caurinus* species.

During our examination of characters we compared both species for the paired cupuliform and countersunk antennal sensilla described by Beutel et al. (2008, fig. 3D) as occurring on the distal part of antennomeres 3 and 4. We found these on antennomeres 4, 5, and 6 (Fig. 7) but could not find them on antennomere 3 of either species. Also, we found the countersunk sensillum but not the cupuliform sensillum. We studied 5 specimens of *Caurinus dectes* and 5 of *Caurinus tlagu*, 3 males and 2 females of each, and were able to see sensilla on 2 female *Caurinus dectes* and 1 male and 2 female *Caurinus tlagu* but on no others. A shorter type of setae with a thicker apex is present near the countersunk sensilla (Fig. 7) which were also visible on those specimens on which we did not find sensilla. This lack of confirmation is likely due to the fixed positioning of the specimens for SEM imaging hiding the sensilla from view, although infraspecific variation and absence cannot yet be eliminated as explanations. The lack of sensilla on antennomere 3 of *Caurinus dectes* raises the possibility that there are multiple species under the name *Caurinus dectes*.

We examined the gonostyles of the males (Fig. 8B) for diagnostic characters. These complex structures may still hold diagnostic potential. In particular, the apex of the gonostyle’s setose basal tooth appeared tapered in *Caurinus tlagu* and truncate in *Caurinus dectes*. However, we were not able to confirm this state was constant in each species. The shape of the upper blade and the pattern of scale-like ridges on the upper blade also appeared to differ. Further study indicated these differences were probably due to differences in the available angles of viewing within the SEM.

We do not know the explanation for the very large COII difference (21.7%) seen between the GenBank *Caurinus dectes* record and our own sequences of seven *Caurinus dectes* specimens. Both samples were made by the same collector, and author of the species, L. Russell, from the type locality. The GenBank record for the *Caurinus dectes* COII is 4.5% different from that of the GenBank record for *Panorpa debilis* (AF424023.1) from the same study (Whiting 2002) which suggests possible contamination or data mixup. Given the ambiguity of the GenBank record’s accuracy we decided to exclude it from our analyses.

The two specimens recovered from the treeless alpine tundra site appear to violate characterizations of *Caurinus* being a forest associated lineage. However, *Caurinus dectes* is often recovered from forested and open rocky sites with the common moss *Rhytidiadelphus loreus*, which represented 20% of the total vegetation at the alpine site (K. LaBounty pers. com.). That *Caurinus tlagu* occurs in clear-cuts and secondary growth sites suggests it is not a habitat specialist. However, within the secondary growth sites in which *Caurinus tlagu* was found, it was significantly more common in thinned sites (n= 11) than in unthinned (n=1). The former have been opened by the Forest Service program TWYGS (Tongass Wide Young Growth Studies) in which the trees have been thinned to encourage old-growth conditions whereas the latter habitats are closed-canopy and dark due to the overcrowding of even-aged trees. This does raise questions about the feeding and breeding ecology of *Caurinus tlagu*. Russell (1979b, 1982) documented *Caurinus dectes* as a specialist on epiphytic and terrestrial leafy liverworts (Jungermanniales). We lack adequate data on the bryophyte communities of the lowland forested sites to assess whether *Caurinus tlagu* shows the same bryophyte associations as *Caurinus dectes*. In particular, seven specimens (19% of our total catch) were found in recently deforested clear cuts, which are likely to have highly disturbed bryophyte communities.

Another notable difference between these *Caurinus* species may be their phenology. Russell (1982) describes adult *Caurinus dectes* as primarily active during the winter (October – April), but reappearing in unseasonably wet, cool weather during the summer. This contrasts with our findings of summer presence of adult *Caurinus tlagu*. Of course, *Caurinus tlagu* could also be active year-round but our sampling regime would fail to detect anything but summer activity.

Various plausible scenarios exist to explain the 1,059 km range disjunction and presumed allopatric speciation within this genus of wingless mecopterans. Either or both populations could be the result of ancient (paleoendemism) or recent (neoendemism) dispersal from the other population or elsewhere (now extinct, or as yet unfound). Such dispersal could be as simple as the ancient transport of *Caurinus*-laden bryophytes by a bird. Given the genetic divergence between the populations, human transport is unlikely because it would be too recent. Alternatively, and we think more likely, both populations may be relicts of an ancient, and much larger population, with subsequent intervening extinction (paleoendemism). A multi-locus population genetics analysis with incorporation of data regarding the region’s geological history would be needed to test these alternatives. Finally, these animals are not easily found and undetected populations may occur in intervening British Columbia.

Prince of Wales Island was mostly buried under an ice sheet during the maximum of the late Wisconsin glaciation 26,000 to 13,000 ^14^C years BP (Carrara et al. 2007) and had been repeatedly buried by ice during the Pleistocene. However, considerable biological and geological evidence suggests that ice-free refugia may have existed during this time, allowing many diverse taxa to continue to evolve in relative isolation, and re-seed the region after deglaciation (Carrara et al. 2007). Of 108 mammal species or subspecies occurring in southeastern Alaska, 27 are endemic to the area (Cook et al. 2001). The known locations of *Caurinus tlagu* are in regions that were reconstructed as under ice by Carrara et al. (2007, fig. 3). Post deglaciation dispersal to these sites from ice-free refugia is the most likely explanation. This suggests, and it would be likely regardless, that *Caurinus tlagu* is more widely distributed than we have documented.

Despite their strong phenotypic similarity, the weight of the evidence supports the conclusion that these separate populations are not conspecific. Their mtDNA sequences being 7.17% divergent (corrected for multiple hits) suggests they have been isolated for probably less than 10 million years (Klicka and Zink 1997, Papadopoulou et al. 2010). Regardless, they have probably been isolated for longer than *Boreus westwoodi* and *Boreus hyemalis* have been isolated from each other. This degree of separation eliminates a late Pleistocene (100,000–250,000 YBP) speciation event hypothesis. The corrected genetic distances between *Boreus* and *Caurinus* (over 103%), indicate the COII gene is fully saturated with multiple hits at this level of comparison, and support the hypothesis of Russell (1979b) that *Caurinus* is a lineage of great age and not an example of relatively recent evolutionary reversal that would make the Boreinae paraphyletic.

This suggests the split between the genus *Caurinus* and the remaining boreids likely predates the oldest confirmed boreid fossil, *Palaeoboreus zherichini* Sukatsheva & Rasnitsyn, of the Late Jurassic (Grimaldi and Engel 2005) which appears to be a boreine due to its size and external ovipositor, although it lacks the produced rostrum typical of extant species (Russell pers. com.). If confirmed, such a great age (>145 Ma) for a genus of two extant species would make the lineage an evolutionary relict and its species certainly deserving of conservation attention (Habel and Assmann 2010, Naskrecki 2011).

## Supplementary Material

XML Treatment for Caurinus tlagu

## Addendum

During 16–17 May 2013, Loren Russell, the author of *Caurinus dectes* and authority on the ecology of the genus, joined us on Prince of Wales to collect and study *Caurinus tlagu*, and show us how to target its host bryophyte. It took us two years (2010 and 2011) to collect 37 *Caurinus tlagu* specimens using three structured sampling methods at 24 sites. In a few hours of collecting, L. Russell was able to collect over a dozen *Caurinus tlagu* and taught us how to brush them from one of their preferred hosts (*Scapania bolanderi*). A video of L. Russell showing this method is available at https://vimeo.com/68819818 and a second video showing *Caurinus tlagu* hopping is available at https://vimeo.com/68819819. Russell also alerted us to an earlier, ecological study that documented *Caurinus* from the Maybeso Experimental Forest on Prince of Wales Island (LeSage et al. 2005). We were able to confirm that voucher specimens of *Caurinus* from this 2005 study are deposited in the Michigan State University collection.
